# Genome-wide association studies identified *OsTMF* as a gene regulating rice seed germination under salt stress

**DOI:** 10.3389/fpls.2024.1384246

**Published:** 2024-03-27

**Authors:** Lifeng Liu, Yanling Ma, Heng Zhao, Lin Guo, Yan Guo, Chun-Ming Liu

**Affiliations:** ^1^Institute of Crop Sciences, Chinese Academy of Agricultural Sciences, Beijing, China; ^2^State Key Laboratory of Plant Environmental Resilience, College of Biological Sciences, China Agricultural University, Beijing, China; ^3^Key Laboratory of Plant Molecular Physiology, Institute of Botany, Chinese Academy of Sciences, Beijing, China; ^4^College of Life Sciences, University of Chinese Academy of Sciences, Beijing, China; ^5^School of Advanced Agricultural Sciences, Peking University, Beijing, China

**Keywords:** rice, seed germination, salt stress, GWAS, *OsTMF*

## Abstract

**Introduction:**

Salt tolerance during seed germination is an important trait for direct seeding and low-cost rice production. Nevertheless, it is still not clear how seed germination under salt stress is regulated genetically.

**Methods:**

In this study, genome-wide association studies (GWAS) were performed to decipher the genetic basis of seed germination under salt stress using 541 rice varieties collected worldwide.

**Results and discussion:**

Three quantitative trait loci (QTLs) were identified including *qGRG3-1* on chromosome 3, *qGRG3-2* on chromosome 5, and *qGRG4* on chromosome 4. Assessment of candidate genes in these loci for their responses to salt stress identified a TATA modulatory factor (*OsTMF*) in *qGRG3-2*. The expression of *OsTMF* was up-regulated in both roots and shoots after exposure to salt stress, and *OsTMF* knockout mutants exhibited delayed seed germination under salt stress. Haplotype analysis showed that rice varieties carrying *OsTMF-Hap2* displayed elevated salt tolerance during seed germination. These results provide important knowledge and resources to improve rice seed germination under salt stress in the future.

## 1 Introduction

Salinity is a major abiotic stress threatening global food production. High salt concentrations in soil cause osmotic stress, ionic toxicity and nutrient deficiency, and hamper plant growth and crop productivity (Hu et al., 2012; Yang and Guo, 2018). For rice production, about 30% agricultural land in the world suffers from high salinity, representing nearly ten thousand hectares in China alone (Takehisa et al., 2004; He and Luo, 2021).

Efforts have been made in the past decades to decipher the mechanisms underlying salt sensitivity in rice through genetic studies. These studies allowed us to identify multiple quantitative trait loci (QTLs) for salt tolerance, although only few genes have been cloned and functionally characterized. *Shoot K^+^ Concentration 1* (*SKC1*) was identified from a F_2_ population generated by crossing the salt-tolerant (ST) *indica* variety ‘Nona Bokra’ with the salt-sensitive (SS) *japonica* variety ‘Koshihikari’. *SKC1* encodes a high-affinity K^+^ transporter that maintains K^+^/Na^+^ equilibrium under salt stress (Lin et al., 2004; Ren et al., 2005). A zinc-finger transcription factor, Drought and Salt Tolerance (DST), regulates salt and drought responses by modulating reactive oxygen species (ROS) homeostasis and stomatal movements (Huang et al., 2009). *Hitomebore Salt Tolerant 1* (*HST1*) was identified as a negative regulator of salt tolerance, and the introduction of a non-functional *hst1* allele to a cultivated variety through backcrosses resulted in improved salt tolerance (Takagi et al., 2015).

With the tremendous progresses made in whole-genome sequencing, genome-wide association studies (GWAS) become a powerful tool to elucidate molecular machinery underlying traits regulated by multiple genes, such as salt tolerance (Wang et al., 2005). In *Arabidopsis*, GWAS was employed to identify genetic components regulating root architecture under salt stress, yielding *Cytochrome P450 family 79 subfamily B2* (*CYP79B2*) as a positive regulator, and *High-Affinity K^+^ Transporter 1* (*HKT1*) as a negative regulator (Julkowska et al., 2017). Zhang et al. (2019) identified *ZmHAK4* that encodes a Na^+^ transporter as a Na^+^ level regulator in maize. In wheat, a QTL in a 1.5 Mb genomic region on chromosome 7B was identified, in which three putative K^+^ transporters (*TaHKT8-B*, *TaHKT9-B* and *TaHKT10-B*) were located. Lines overexpressing *TaHKT9-B* and varieties carrying the In‐1077 haplotype of *TaHKT9-B* displayed higher salt sensitivity due to lower K^+^ accumulation in shoots (Du et al., 2024). Recently, two genes were identified *via* GWAS, a transcription factor OsWRKY53 that regulates water contents in shoots, and a mitogen-activated protein kinase kinase 10.2 (OsMKK10.2) that is implicated in seedling survival under salt stress (Yu et al., 2023).

Seed germination is a critical developmental stage for plants to start a new phase of life. Under salt stress, seed germination in most crop species is compromised, manifested primarily by lower germination rates and longer germination time (Shi et al., 2017; Hasseb et al., 2022; Yang et al., 2023). Increased osmotic pressures induced by salt stress may affect water absorption, starch mobilization and catabolism of storage products during seed germination (Xu et al., 2017; Yan et al., 2022; Xiong et al., 2024). Enzymatic and antioxidant activities, hormone levels and ion homeostasis are also affected by salt stress, which may consequently impact both seed germination and seedling establishment (Munns and Tester, 2008; Yang and Guo, 2018; He et al., 2019).

In recent years, direct seeding is increasingly used in many rice-growing areas because of lower labor input, which demands uniform seed germination and high seedling vigor, especially under stress conditions (Farooq et al., 2011). A QTL for seed germination and seedling establishment under salt stress, *Seedling Establishment 3* (*qSE3*), was identified using substitution lines generated by introgressions of chromosomal segments from a *japonica* variety ‘Jiucaiqing’ (with low seedling establishment under salt stress) into an *indica* variety ‘IR26’ (with high seedling establishment under salt stress). High-resolution mapping revealed that *High-Affinity K^+^ Transporter 21* (*OsHAK21*) is likely to be the candidate gene in *qSE3*, which confers higher salt tolerance by decreasing ROS levels in abscisic acid (ABA)-dependent manner (He et al., 2019). Beyond this, very little is known about the genetic machinery underlying rice seed germination under salt stress.

The aim of this study is to characterize the genetic basis of rice seed germination under salt stress conditions, with the long-term goal to improve seed germination for direct seeding in saline land. We conducted GWAS to dissect the process using a panel of 541 rice varieties collected from different regions of the world. Three QTLs, *qGRG3-1* on chromosome 3, *qGRG3-2* on chromosome 5 and *qGRG4* on chromosome 4, were detected under treatments of two concentrations of NaCl. *OsTMF* was showed to be the candidate gene in *qGRG3-2* as its expression was highly responsive to salt stress, and *ostmf* mutants exhibited delayed seed germination under salt stress.

## 2 Materials and methods

### 2.1 Plant materials

A total of 541 accessions of rice (*Oryza sativa* L.) from major rice-growing areas worldwide, consisting of 328 *indica*, 162 *japonica*, 18 *admix*, 17 *aus*, and 16 *basmati* varieties (**Supplementary Table S1**), were obtained from the China Rice Data Center and the 3,010 Rice Genomes Project (Wang et al., 2018). All varieties were planted, and seeds were harvested in Lingshui, Hainan Province, China. A single nucleotide polymorphism (SNP) dataset was downloaded from the Rice SNP-Seek database (https://snpseek.irri.org/) (Mansueto et al., 2016). Non-sequenced varieties were sequenced on an Illumina HiSeq 2000 Sequencing Platform (Illumina Inc., San Diego, CA, USA), with an average sequencing depth of approximately 10×.

### 2.2 Evaluations of seed germination under salt stress

Fully mature seeds were harvested in field right after the grains turned to yellow, and dried in a 37°C oven for 7 days to break dormancy. Fifty rice varieties (**Supplementary Table S2**) were randomly chosen for germination test to determine the optimal salt stress conditions using 0.3%, 0.4%, 0.5%, 0.6%, 0.7%, 0.8%, 0.9% and 1.0% NaCl (w/v) dissolved in deionized water. Accordingly, the concentrations of 0.3% and 0.5% were subsequently used for GWAS. For each variety, 90 seeds were divided into three replicates and placed onto two layers of filter paper soaked with either 8 mL of deionized water, or 0.3% or 0.5% NaCl solutions, in 6-cm petri dishes without seal. All solutions were refreshed every two days. These dishes were cultured in an incubator at 28°C under a 12 h light/12 h dark photoperiod for 7 days. Germinated seeds, with the criterion of root lengths longer than the seed length, and shoot lengths longer than half of the seed length (Shi et al., 2017), were counted to calculate their germination rates on the 3^rd^ day (GR3), germination rates on the 4^th^ day (GR4), and germination indexes in 7 days (GI), with the calculation formula of GR3 = accumulated number of germinated seeds on the 3^rd^ day/the total number of seeds used × 100%; GR4 = accumulated number of germinated seeds on the 4^th^ day/the total number of seeds used × 100%; GI = ∑(G_t_/T_t_), where G_t_ is the number of germinated seeds on day t and T_t_ is the time in days corresponding to G_t_ (Shi et al., 2017). To obtain germination rate grades on the 3^rd^ (GRG3) and the 4^th^ day (GRG4), and germination index grades (GIG), the ratios of GR3, GR4 or GI for salt treatment to those in the control condition were calculated, and then assigned to germination grades from 1 to 11 (**Supplementary Table S3**). For seed germination in saline soil, pots with the size of 7 cm × 7 cm × 10 cm were filled with soil, and immersed in either deionized water or 0.5% NaCl solution for 24 hours to ensure saturation. Thirty seeds were sown into each pot and covered with a thin layer of soil. Subsequently, these pots were placed in an incubator at 28°C under 12 h light/12 h dark, watered with either deionized water or 0.5% NaCl solution in every two days. The germinated rates and shoot lengths were measured on the 10^th^ day after sowing.

### 2.3 GWAS and haplotype analyses

The general linear model (GLM) with the first three principal components matrix calculated from genotypes was employed using Tassel 5.2.54 software (Bradbury et al., 2007) to perform GWAS for seed germination under salt stress. Across the whole panel of varieties, 302,900 high-quality SNPs were used for GWAS after removing SNPs with missing rates of more than 20% and minor allele frequencies of less than 5% (Ju et al., 2022; Mei et al., 2022). To identify significant SNPs, the Bonferroni correction method was used to calculate suggestive significance thresholds of associations, resulting in a *p* value of 1.65 × 10^-7^ (0.05/N, with N = number of SNPs) (Shi et al., 2017). The qqman package in R3.4.1 software was used to generate Manhattan plots (Turner, 2014). Functional annotations for genes within genomic regions of interest were obtained in reference to the Nipponbare genome IRGSP 1.0 (Kawahara et al., 2013). Haplotype analyses were performed using SNPs in the coding sequences, with each haplotype possessing at least twenty rice varieties (Mei et al., 2022).

### 2.4 qRT-PCR analyses

Candidate genes were investigated by quantitative real-time PCR (qRT-PCR) using RNA extracted from shoots and roots of 7-day-old Zhonghua11 (a *japonica* rice variety, ZH11) seedlings grown under 0.3% or 0.5% NaCl, or control (deionized water) conditions. To examine expression levels of candidate genes in varieties with different germination grades, shoots from 7-day-old seedlings of ST (L_363, L_417 and L_505, with GRG3 and GRG4 ≤ 2) and SS varieties (L_065, L_274 and L_352, with GRG3 and GRG4 ≥9) grown under 0.5% NaCl or control conditions were collected and analyzed by qRT-PCR. To examine expression patterns of *OsTMF* in time-course salt stress treatments, germinated seeds under 0.5% NaCl or control conditions for 1, 2, or 3 days, and seedlings grown in Hoagland solution treated with or without 0.5% NaCl for 1, 3, or 7 days, were sampled. Total RNA was extracted using TRIzol™ reagent (Thermo Fisher, USA), cDNA was synthesized using the PrimeScript™ RT reagent kit (Takara, Japan), and qRT-PCR was conducted using TB Green^®^ Premix Ex Taq™ II (Takara, Japan) on LightCycler^®^ 96 (Roche Life Science, Switzerland). Primers for qRT-PCR were designed using Primer3 (https://primer3.ut.ee) based on coding sequences of genes (**Supplementary Table S4**). *OsActin1* was used as an internal control for normalization, and the 2^−△△CT^ method was used to calculate relative expression levels.

### 2.5 Generations of knockout mutants using CRISPR/Cas9

Clustered regularly interspaced short palindromic repeat (CRISPR)/CRISPR-associated nuclease 9 (CRISPR/Cas9) constructs were made according to Cheng et al. (2021), using the guide sequences for candidate genes designed by the web tool CRISPR-GE (http://cbi.hzau.edu.cn/cgi-bin/CRISPR) (Xie et al., 2017; **Supplementary Table S5**), and transformed into ZH11 *via Agrobacterium*-mediated transformation (Nishimura et al., 2007). Sanger sequencing (Sangon Biotech, Beijing) was performed to identify homozygous mutants. PCR products amplified with *HPT*-F (5′-TGCCGTCAACCAAGCTCTGA-3′) and *HPT*-R (5′-GCTTCGATGTAGGAGGGCGT-3′) primers were used to select transgene-free lines.

### 2.6 Statistical analysis

Correlation analyses and significant differences were assessed between pairs of evaluated traits using SAS software (Shi et al., 2017). Differences between varieties, subpopulations, treatments, or haplotypes were examined using a one-way ANOVA method based on a significance level of 0.01. Figures were generated using GraphPad Prism 8.0 software (https://www.graphpad.com).

## 3 Results

### 3.1 Phenotypic variations of rice varieties for seed germination under salt stress

To define optimal conditions for assessing rice seed germination under salt stress, seeds of 50 varieties randomly selected from 541 varieties collected from different regions of the world (**Supplementary Table S2**) were germinated in either deionized water (control) or different concentrations of NaCl solutions (see Materials and Methods). Well-germinated seeds, according to the criterion reported (Shi et al., 2017) were counted every day over a 7-day period. GRG3, GRG4 and GIG were calculated, with germination grade 1 for the highest, and grade 11 for the lowest salt tolerances (see Materials and Methods). Results showed that, under 0.3% and 0.5% NaCl treatments, all three traits were consistently distributed (**Supplementary Figure S1**), therefore, these two treatments were used for subsequently experiments.

**Figure 1:**
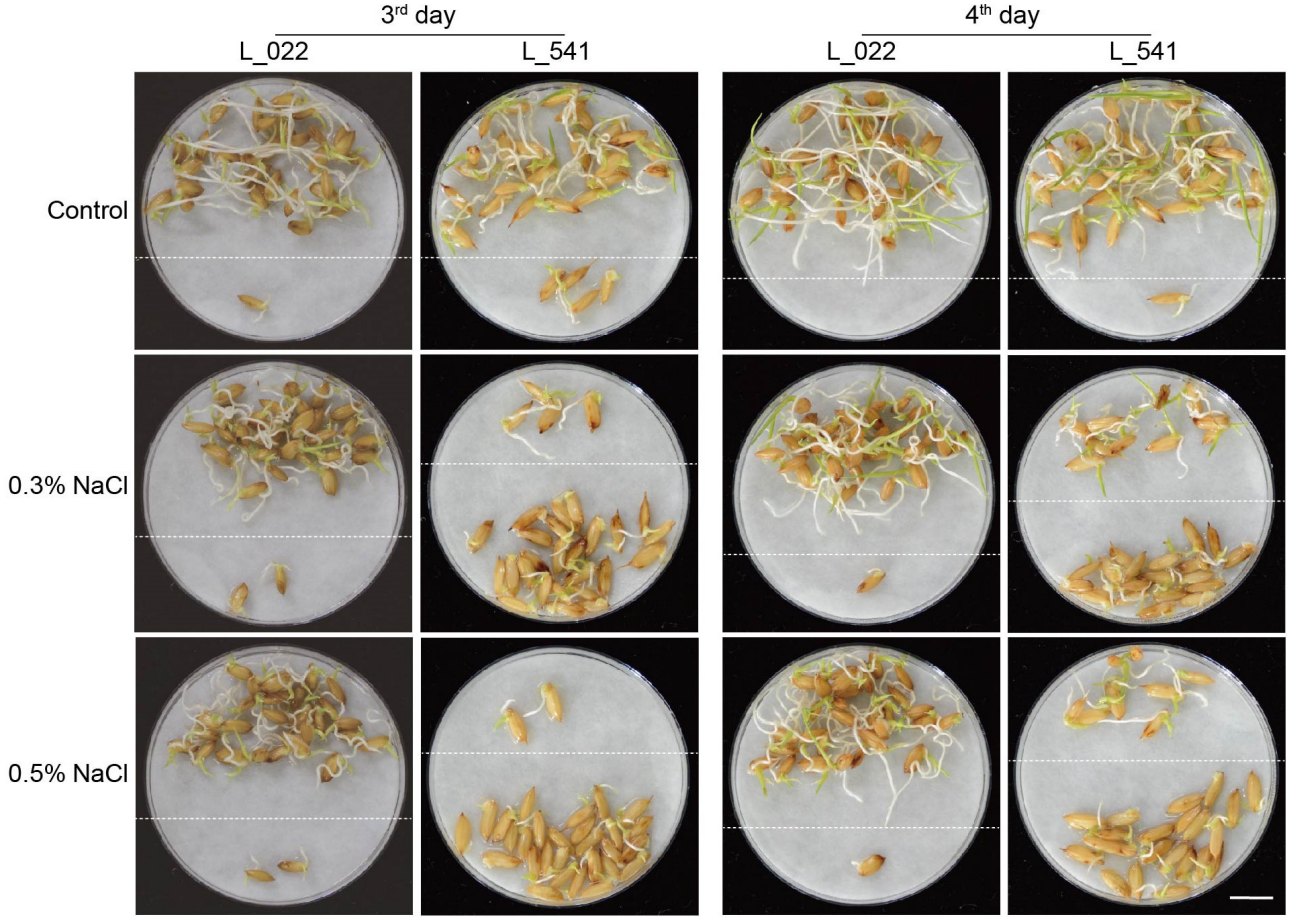
Differences in seed germination under salt stress in two representative rice varieties. Representative photos of rice seed germinations under salt stress, on the 3^rd^ and 4^th^ days for the salt-tolerant (ST) variety L_022 and the salt-sensitive (SS) variety L_541. Seeds were placed on filter paper soaked with deionized water (control), 0.3% or 0.5% NaCl for the indicated numbers of days. Germinated seeds, with root length longer than seed length and shoot length longer than half of the seed length, were repositioned above dashed lines. Scale bar, 1 cm.

**Figure 2:**
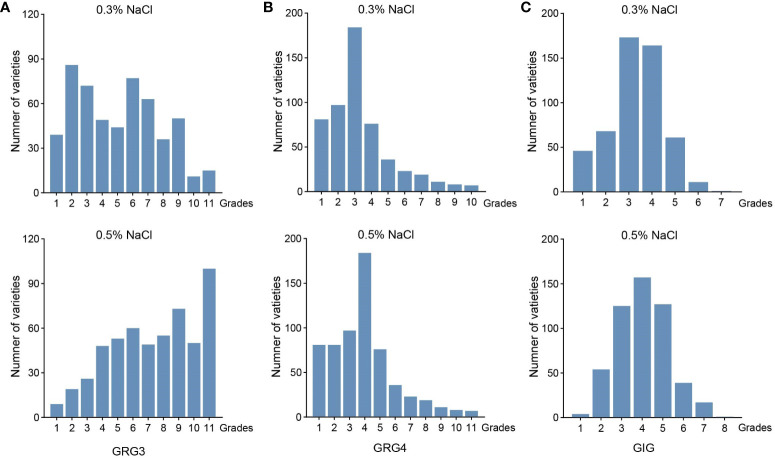
Phenotypic variations of 541 rice varieties in seed germinations under salt stress. **(A)** Germination rate grades on the 3^rd^ day (GRG3) under the 0.3% (top) and 0.5% NaCl treatments (bottom). **(B)** Germination rate grades on the 4^th^ day (GRG4) under the 0.3% (top) and 0.5% NaCl treatment (bottom). **(C)** Germination index grades (GIG) under the 0.3% (top) and 0.5% NaCl treatments (bottom).

To examine variations in seed germinations under salt stress in 541 rice varieties, seeds of these varieties were placed on plates with water, 0.3% or 0.5% NaCl, and germinations were counted daily over a 7-day period. Variations of seed germinations under salt stress were observed in different varieties (**Supplementary Table S1**). As showed for two representative varieties, L_541 with lower salt tolerance, and L_022 with higher salt tolerance in germination, numbers of germinated seeds differed on the 3^rd^ and the 4^th^ day, with more germinated seeds for the ST variety (**Figure 1**; above dash lines). All three traits, GRG3, GRG4 and GIG, showed large variations (**Figure 2**), with the highest coefficient of variation observed for GRG4 (73.53%) under 0.3% NaCl condition, followed by GRG4 under 0.5% NaCl (57.31%), and GRG3 under 0.3% NaCl (53.43%) (**Table 1**). Phenotypic variations in subpopulations were also examined, and results showed that, under treatments of either 0.3% or 0.5% NaCl, *indica* varieties displayed lower GRG3 (**Supplementary Figure S2A**) and GRG4 (**Supplementary Figure S2B**) when compared with other 4 subpopulations that showed no significant differences, suggesting a higher salt tolerance of *indica* varieties. To be noted, no significant difference was observed for GIG among these subpopulations (**Supplementary Figure S2C**). In addition, on average, GRG3 was higher than GRG4 under both 0.3% and 0.5% NaCl (**Table 1**), suggesting that the germination of rice seeds on the 3^rd^ day was more vulnerable to salt stress than that on the 4^th^ day. Further analyses revealed that GRG3 and GRG4 had moderately positive correlations at these two NaCl concentrations (**Supplementary Table S6**).

**Table 1:** Results of seed germinations under salt stresses in 541 rice varieties.

Treatments	Traits	Germination grade ranges	Mean	Standard deviations	Coefficient of variations
0.3% NaCl	Germination rate grades on the 3^rd^ day (GRG3)	1–11	5.0832	2.7160	53.43%
	Germination rate grades on the 4^th^ day (GRG4)	1–10	2.7116	1.9940	73.53%
	Germination index grades (GIG)	1–7	3.3117	1.1779	35.57%
0.5% NaCl	Germination rate grades on the 3^rd^ day (GRG3)	1–11	7.3401	2.8013	38.16%
	Germination rate grades on the 4^th^ day (GRG4)	1–11	3.3346	1.9111	57.31%
	Germination index grades (GIG)	1–8	4.0286	1.2599	31.27%

### 3.2 QTLs associated with seed germination under salt stress

**Figure 3:**
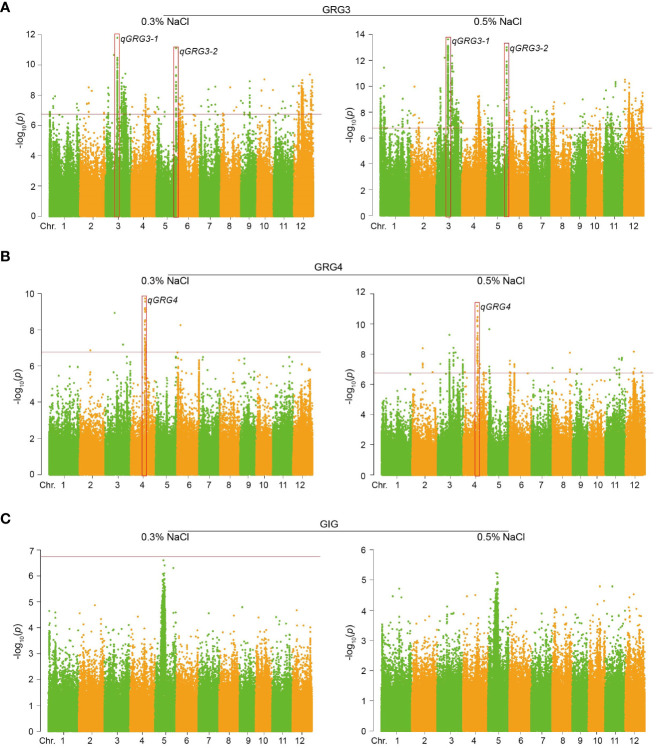
Manhattan plots for GWAS of rice seed germinations under salt stress. **(A)** Manhattan plots of GRG3 under 0.3% (left) or 0.5% NaCl treatments (right). **(B)** Manhattan plots of GRG4 under 0.3% (left) or 0.5% NaCl treatments (right). **(C)** Manhattan plots of GIG under 0.3% (left) or 0.5% NaCl treatments (right). Red boxes show genomic regions with clustered SNPs above the significance thresholds (−log10(*p*); indicated by horizontal red lines).

To dissect the genetic basis of rice seed germination under salt stress, GWAS was conducted based on the GLM model using 302,900 SNPs from public database and in-house re-sequencing project. These SNPs were evenly distributed across all 12 chromosomes, with a density of about 800 SNPs/Mb (**Supplementary Table S7**). The significance threshold for GWAS was set to 6.78 (−log_10_(0.05/N), N = effective SNP number) after Bonferroni correction. Manhattan plots were generated, showing that 1,494 SNPs were significantly correlated with rice seed germination under salt stress, in particular, GRG3 (**Figure 3A**; **Supplementary Table S8**) and GRG4 (**Figure 3B**; **Supplementary Table S8**). These SNPs explained 4.54% to 10.06% of phenotypic variations. No SNP association was identified for GIG (**Figure 3C**).

To identify putative genomic regions associated with salt tolerance, we screened for co-located and clustered SNPs detected under both 0.3% and 0.5% NaCl treatments. Three QTLs, *qGRG3-1* (chr.3; 16,666,490 − 17,013,993) and *qGRG3-2* (chr.5; 27,858,716 − 28,081,822) associated with GRG3 (**Figure 3A**), and *qGRG4* (chr.4; 19,891,019 − 20,069,206) associated with GRG4 (**Figure 3B**), were identified. Notably, the *qGRG4* locus contained a high-affinity potassium transporter *OsHAK1*, harboring a significant SNP (S04_19891019, −log_10_(*p*) = 7.19 under 0.3% and −log_10_(*p*) = 7.58 under 0.5% NaCl). Since *OsHAK1* is known to regulate K^+^/Na^+^ homeostasis in rice seedlings under salt stress (Chen et al., 2015), our subsequent work was focused on two other loci, *qGRG3-1* and *qGRG3-2*.

### 3.3 Identification of candidate genes responsive to salt stress

**Figure 4:**
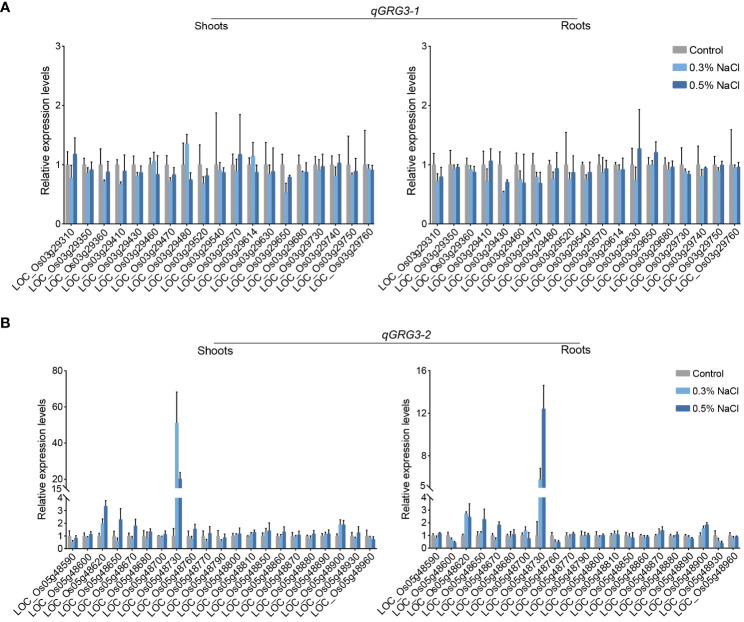
Relative expressions of genes in *qGRG3-1* and *qGRG3-2* loci under the salt stress. qRT-PCR analyses of genes in *qGRG3-1* **(A)** and *qGRG3-2* **(B)** loci in shoots (left) and roots (right) excised from 7-day-old ZH11 seedlings germinated under control (deionized water), 0.3% NaCl or 0.5% NaCl conditions. Data are shown as means ± SD from three biological replicates.

To identify candidate genes that are responsive to salt stress in *qGRG3-1* and *qGRG3-2* loci, the roots and shoots of 7-day-old ZH11 seedlings grown under 0.3% or 0.5% NaCl treatments were collected, and expression analyses were performed using qRT-PCR to analyze expressions of genes located in these two loci. No consistent responses to salt stress were observed in any of the 19 genes in the *qGRG3-1* locus (**Figure 4A**). In contrast, 8 of 21 genes examined in the *qGRG3-2* locus showed responsive expressions under the salt stress (**Figure 4B**). In particular, LOC_Os05g48620, LOC_Os05g48730, and LOC_Os05g48900 were up-regulated in roots and shoots of seedlings exposed to either 0.3% or 0.5% NaCl, while expressions of LOC_Os05g48650 and LOC_Os05g48670 were only up-regulated under 0.5% NaCl treatment (**Figure 4B**). Three other genes, LOC_Os05g48600, LOC_Os05g48760 and LOC_Os05g48930, were down-regulated in roots upon exposure to 0.3% or 0.5% NaCl (**Figure 4B**, right). We selected these genes as candidates for further analysis.

We examined expressions of these eight candidate genes in three ST (L_363, L_417, and L_505) and three SS varieties (L_065, L_274, and L_352), selected as described in Materials and Methods. Results showed that LOC_Os05g48730 (**Supplementary Figure S3A**) and LOC_Os05g48930 (**Supplementary Figure S3B**) exhibited higher levels of expression in ST varieties than those in SS varieties, under both the control and salt stress conditions. Three other genes, LOC_Os05g48620 (**Supplementary Figure S3C**), LOC_Os05g48670 (**Supplementary Figure S3D**) and LOC_Os05g48760 (**Supplementary Figure S3E**), showed elevated expressions under salt stress in ST varieties, but not in SS varieties. For LOC_Os05g48600, elevated expressions were observed in L_363 (ST) and L_417 (ST) under salt stress, but no significantly changed expressions in L_505 (ST), and three SS varieties (L_065, L_274 and L_352; **Supplementary Figure S3F**). For LOC_Os05g48650 and LOC_Os05g48900, although no consistent pattern was observed between ST and SS varieties, elevated expressions were detected for LOC_Os05g48650 under salt stress in all varieties except L_065 (**Supplementary Figure S3G**), whereas no salt-induced expressions were observed for LOC_Os05g48900 in neither ST nor SS varieties (**Supplementary Figure S3H**).

### 3.4 Genetic characterization of genes underlying seed germination under salt stress

**Figure 5:**
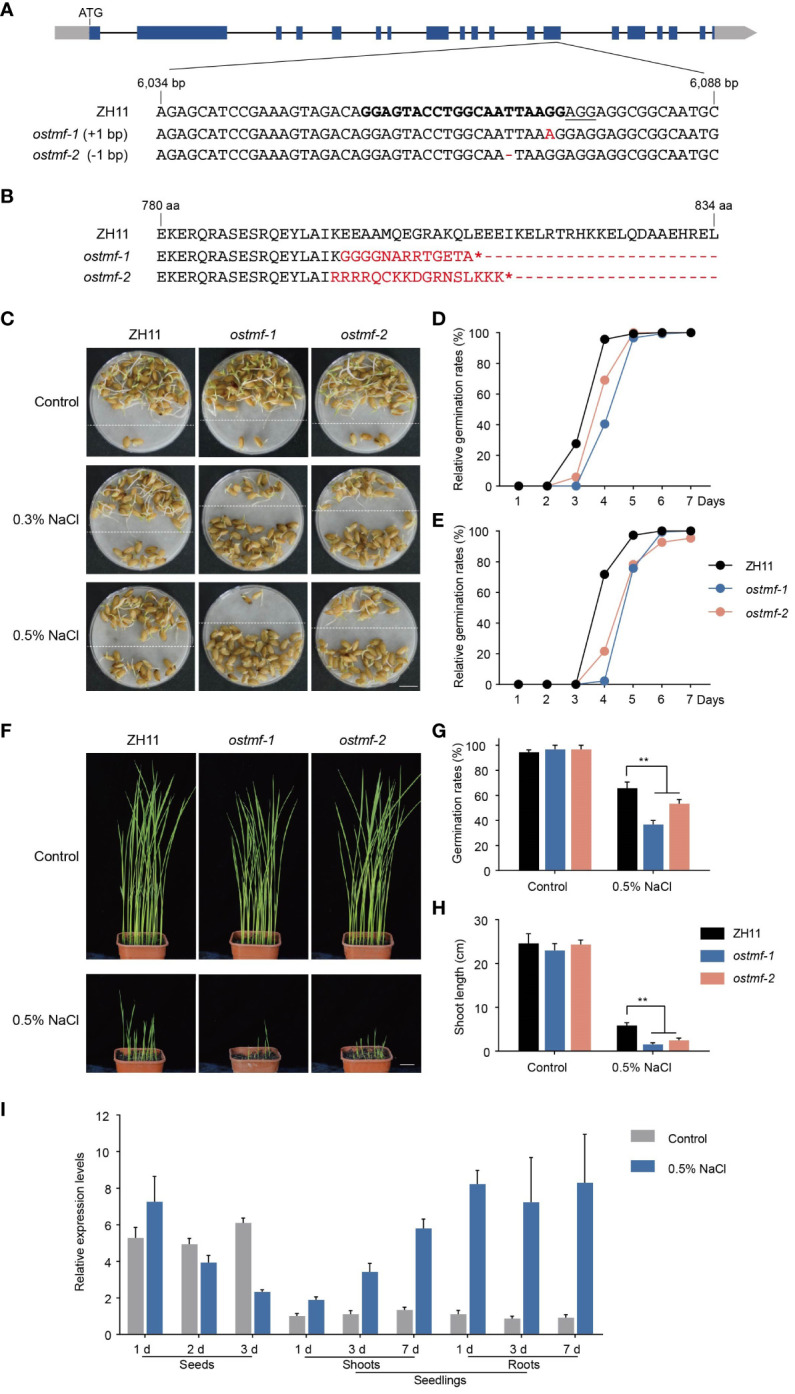
Mutations of *OsTMF* led to delayed seed germinations under salt stress. **(A)** A diagram of the *OsTMF* gene model (top), and partial sequence of *OsTMF* containing the target sites (in bold) for CRISPR/Cas9-based gene editing in ZH11, and the sequences of *ostmf-1* and *ostmf-2* mutants. Blue rectangles represent exons, black lines represent introns, and gray rectangles represent untranslated regions. The protospacer adjacent motif (PAM) is underlined, and mutations are in red. **(B)** Partial peptide sequences of OsTMF from ZH11, *ostmf-1* and *ostmf-2*. Red, altered sequences in *ostmf-1* and *ostmf-2*; asterisks, stop codon. **(C)** Representative photos for seed germinations of ZH11 and *ostmf* mutants under the control (deionized water), 0.3% or 0.5% NaCl treatments for 4 days. Germinated seeds were repositioned above the dashed lines. Scale bar, 1 cm. **(D, E)** Relative germination rates of ZH11 and *ostmf* mutants in different time points under 0.3% **(D)** and 0.5% **(E)** NaCl treatments. **(F)** Ten-day-old seedlings of ZH11 and *ostmf* mutants, germinated in soil-filled pots immersed in deionized water (the upper panel) or 0.5% NaCl (the lower panel). Scale bar, 2 cm. **(G)** Germination rates on the 10^th^ day of ZH11 and *ostmf* mutants. germinated in soil-filled pots immersed in deionized water or 0.5% NaCl. **, *p* < 0.05. **(H)** Shoot length of seedlings of 10-day-old ZH11 and *ostmf* mutants germinated in soil-filled pots immersed in deionized water or 0.5% NaCl **, *p* < 0.05. **(I)** Expressions of *OsTMF* in seeds (treated with deionized water or 0.5% NaCl for 1, 2 or 3 days) and seedlings (treated with Hoagland solution or Hoagland solution containing 0.5% NaCl for 1, 3 or 7 days).

To further characterize the eight candidate genes in *qGRG3-2* locus, knockout mutants were generated using CRISPR/Cas9-mediated gene editing in ZH11. For each gene, at least two independent homozygous transgene-free lines with frameshifts in translation were identified in either T1 or T2 generations, confirmed by PCR amplifications and sequencing (**Supplementary Table S5**). We tested the germination rates of mature seeds from mutant lines under the control, 0.3% and 0.5% NaCl treatments. Among lines assayed, *ostmf-1* and *ostmf-2* (**Figures 5A, B**), carrying mutations in LOC_Os05g48620 that encodes OsTMF (a rice homolog of human TATA modulatory factor, TMF), exhibited delayed germination under 0.3% NaCl treatments when compared to ZH11 (**Figures 5C, D**). OsTMF was previously reported to attenuate cold tolerance in rice by modulating expressions of cell wall biosynthesis-related genes (Xu et al., 2020). With the increased NaCl concentration (0.5%), further delays of germination were observed (**Figures 5C** (below dashed lines), **E**).

To examine the germinations of *ostmf-1* and *ostmf-2* in saline soil, we sowed their seeds in pots, filled with soil, and soaked in deionized water or 0.5% NaCl. On the 10^th^ day, no evident phenotypic difference was observed in *ostmf-1* and *ostmf-2* under the control conditions, when compared to ZH11 (**Figure 5F**). However, in soil soaked with 0.5% NaCl, both *ostmf-1* and *ostmf-2* showed lower germination rates (**Figure 5G**) and shorter shoot lengths (**Figure 5H**).

To investigate the expression of *OsTMF* in response to salt stress, we conducted qRT-PCR analyses in seeds and seedlings using RNAs collected at different days after growing in 0.5% NaCl and deionized water. Results showed that the *OsTMF* expression was increased in seeds after growing in 0.5% NaCl solution for 1 day, and then decreased gradually, whereas it maintained stable in those seeds grown in water (**Figure 5I**). In the shoots and roots of seedlings, salt stress enhanced *OsTMF* expressions from day 1 to 7, whereas in the control conditions, *OsTMF* expression remained at stable and low levels throughout the time course. These findings suggest that *OsTMF* may positively regulate seed germination under salt stress.

### 3.5 Haplotype analysis of *OsTMF*

**Figure 6:**
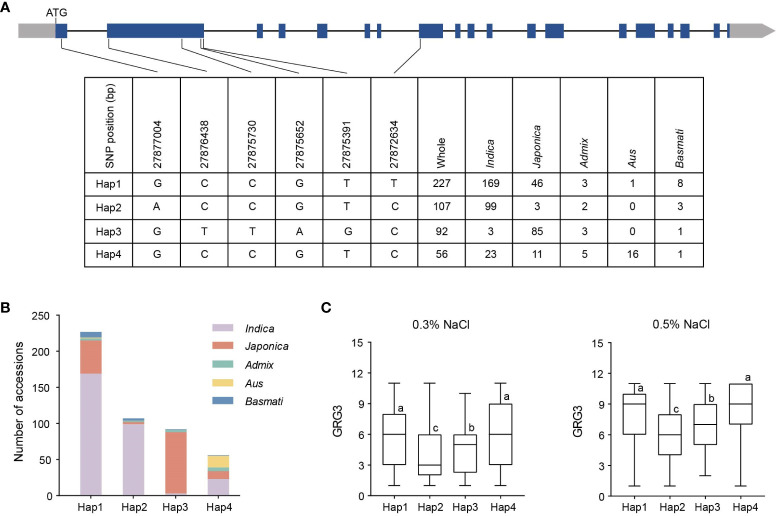
Haplotype analyses in *OsTMF*. **(A)** Genomic variations in *OsTMF* coding region, showing the presence of 4 haplotypes (Hap) in 541 rice varieties. **(B)** Frequencies of 4 *OsTMF* haplotypes in different rice subpopulations. **(C)** Comparison of GRG3 under 0.3% (left) or 0.5% NaCl (right) in 4 *OsTMF* haplotypes. Lowercase letters indicate significant differences based on Duncan’s multiple range post-hoc test (*p* < 0.05).

To examine the relationship between *OsTMF* haplotypes and seed germination under salt stress, haplotype analysis was conducted in 541 rice varieties. In the coding region of *OsTMF*, six SNPs were detected (**Figure 6A**), with which we defined four major haplotypes, based on their presences in at least 20 varieties. We observed that Hap1, Hap2, and Hap4 are predominant present in the *indica* subpopulation, while Hap3 is predominant in the *japonica* subpopulation (**Figures 6A, B**). GRG3 of varieties harboring Hap2 were 3.710 and 5.794 under 0.3% and 0.5% NaCl treatments, respectively, which were lower than those varieties carrying Hap1, Hap3, or Hap4 (**Figure 6C**), indicating that Hap2 is more tolerant to salt stress. These results together suggest that Hap2 is a potential haplotype to be used for improving rice seed germination under salt stress.

## 4 Discussion

Efficient seed germination under salt stress is a crucial prerequisite for seedling establishment and subsequent crop production in saline land, and is a quantitative trait regulated by multiple genes (Shi et al., 2017; Li et al., 2022). In this study, we conducted GWAS to investigate the genetic basis of seed germination under salt stress utilizing a set of 541 rice varieties collected worldwide. We identified three QTLs, *qGRG3-1*, *qGRG3-2* and *qGRG4*, which are significantly associated with seed germination under salt stress, imposed by using 0.3% or 0.5% NaCl. Detailed analyses showed that the most likely candidate gene underlying *qGRG3-2* is *OsTMF*. Under salt stress, the expressions of *OsTMF* were up-regulated, and *ostmf* mutants exhibited delayed seed germinations when compared to the wild type.

In general, rice seed germination is defined visually by the emergence of the radicle penetrating through the hull, which has been used to study seed dormancy and germination (Jung et al., 2019; Prasad et al., 2022; Jin et al., 2023). However, under salt stress, rice seeds exhibit not only delayed germination, but also compromised growths of radicle and coleoptile, leading to lower seedling viability. Therefore, a more adequate criterion to define seed germination under salt stress is based on a combination of two measures: the root length longer than the seed length, and the shoot length longer than half of the seed length (Shi et al., 2017). To accurately measure seed germination in such a manner, we did the germination experiments on water- or NaCl solution-immersed filter paper in petri dishes (with a cover, but without seal), and refreshed the solutions in every two days. After counting germinated seeds, germination rate and germination index were calculated to evaluate seed germination status under salt stress (Shi et al., 2017; Ju et al., 2022; Li et al., 2022; Mei et al., 2022; Zhan et al., 2023). In this study, we established germination grades (1 to 11) using the GR3, GR4 and GI ratios of salt stress condition to the control condition. Under such a criterion, all varieties showed continuous and extensive phenotypic variations, suggesting that they are suitable for GWAS analysis.

Although GWASs have been performed in rice to decipher seed germination under salt stress (Shi et al., 2017; Yu et al., 2018; Islam et al., 2022; Ju et al., 2022; Li et al., 2022), no genes have been identified so far. In this study, three QTLs, two for GRG3 (*qGRG3-1* and *qGRG3-2*) and one for GRG4 (*qGRG4*), were consistently detected under 0.3% and 0.5% NaCl treatments. In the *qGRG4* locus, *OsHAK1*, a well-characterized high-affinity K^+^ transporter that promotes rice seedling growth under salt stress, is present (Chen et al., 2015, 2018). The *OsHAK1* gene was also reported before in the *q4.8* locus (Zhang et al., 2023), and is associated with relative germination rate on the 3^rd^ day under saline-alkali stress. In addition, other HAK genes such as *OsHAK12* (Zhang et al., 2021), *OsHAK16* (Feng et al., 2019) and *OsHAK21* (He et al., 2019; Song et al., 2021) are implicated in salt tolerance in rice. Among them, *OsHAK21* may play a role in seed germination under salt stress (He et al., 2019). Therefore, in experiments followed, we did not pay much attention to the genomic region of *qGRG4*. *qGRG3-1* overlaps with the reported 16.66−17.02 Mbp region on chromosome 3, which is associated with shoot fresh weight under salt stress (Yu et al., 2023). *qGRG3-2* overlaps with the reported *q5.11* locus that is associated with the relative germination rate under saline-alkali stress (Zhang et al., 2023). However, no salt tolerance-related gene has been reported in these regions.

Expression analysis is a powerful way to identify genes underlying salt tolerance, with either elevated (Chen et al., 2018; Feng et al., 2019) or repressed expressions under salt stress (Wang et al., 2020), or differential expressions in ST and SS varieties (Zhang et al., 2018, 2019). We examined expressions of genes located in *qGRG3-1* and *qGRG3-2* loci, which allowed us to identify eight candidate genes in the *qGRG3-2* locus, which exhibited elevated or repressed expressions under salt stresses. Among them, five showed differential expressions between SS and ST varieties. To narrow down candidate genes in the *qGRG3-2* locus, we employed CRISPR/Cas9-based gene editing to generate mutants for all these eight genes. Seed germination analyses performed in these mutants under salt stress allowed us to establish that *OsTMF* is the most likely candidate gene, since *OsTMF* knockout mutants showed delayed seed germination under salt stress. We showed further that varieties with different *OsTMF* haplotypes showed differences in early seed germination under salt stress. However, as the germination progressed to the seventh day, the germination rates of *ostmf* mutants were comparable with ZH11, suggesting a major role of *OsTMF* in regulating early seed germination under salt stress, not so much in seedling establishment. *OsTMF* has previously been reported to be a negative regulator of cold tolerance in rice (Xu et al., 2020). In another report, it was shown that OsTMF interacted with Ski-Interacting Protein a (OsSKIPa), that regulates cell vitality, and overexpression of *OsSKIPa* led to enhanced vegetative growth under salt stress (Hou et al., 2009). Whether OsTMF acts through OsSKIPa in seed germination under salt stress warrants further investigation.

## Data availability statement

The datasets presented in this study can be found in online repositories. The names of the repository/repositories and accession number(s) can be found in the article/**Supplementary Material**.

## Author contributions

LL: Conceptualization, Data curation, Formal Analysis, Investigation, Methodology, Software, Writing – original draft, Writing – review & editing. YM: Data curation, Writing – review & editing. HZ: Writing – review & editing. LG: Investigation, Writing – review & editing. YG: Supervision, Validation, Writing – review & editing. C-ML: Formal Analysis, Funding acquisition, Project administration, Resources, Supervision, Validation, Visualization, Writing – original draft, Writing – review & editing.

## Supplementary material

Supplementary Figure 1 Phenotypic variations of 50 randomly selected rice varieties for seed germination in NaCl concentrations from 0.3% to 1%. Germination rate grades on the 3^rd^ (GRG3; **A**) and the 4^th^ day (GRG4; **B**), and germination index grades (GIG; **C**) under the treatments if indicated NaCl concentrations.

Supplementary Figure 2 Phenotypic variations for seed germinations under salt stresses among varieties in different subpopulations. **(A)** Germination rate grades on the 3^rd^ day (GRG3) under 0.3% (top) and 0.5% NaCl (bottom) treatments in different subpopulations. **(B)** Germination rate grades on the 4^th^ day (GRG4) under 0.3% (top) and 0.5% NaCl (bottom) conditions in different subpopulations. **(C)** Germination index grades (GIG) under the treatment of 0.3% (top) and 0.5% NaCl (bottom) in different subpopulations. Lowercase letters indicate significant differences based on Duncan’s multiple range post-hoc test (*p* < 0.05).

Supplementary Figure 3 Expressions of candidate genes in the *qGRG3-2* locus in representative salt-tolerant and salt-sensitive varieties. ST, representative salt-tolerant varieties; SS, representative salt-sensitive varieties.
