# Supplementary figures and images for: Genome-wide association studies identified OsTMF as a gene regulating rice seed germination under salt stress
Source: Front Plant Sci. 2024 Mar 27;15:1384246. doi: 10.3389/fpls.2024.1384246 (PMC11004275; doi:10.3389/fpls.2024.1384246)

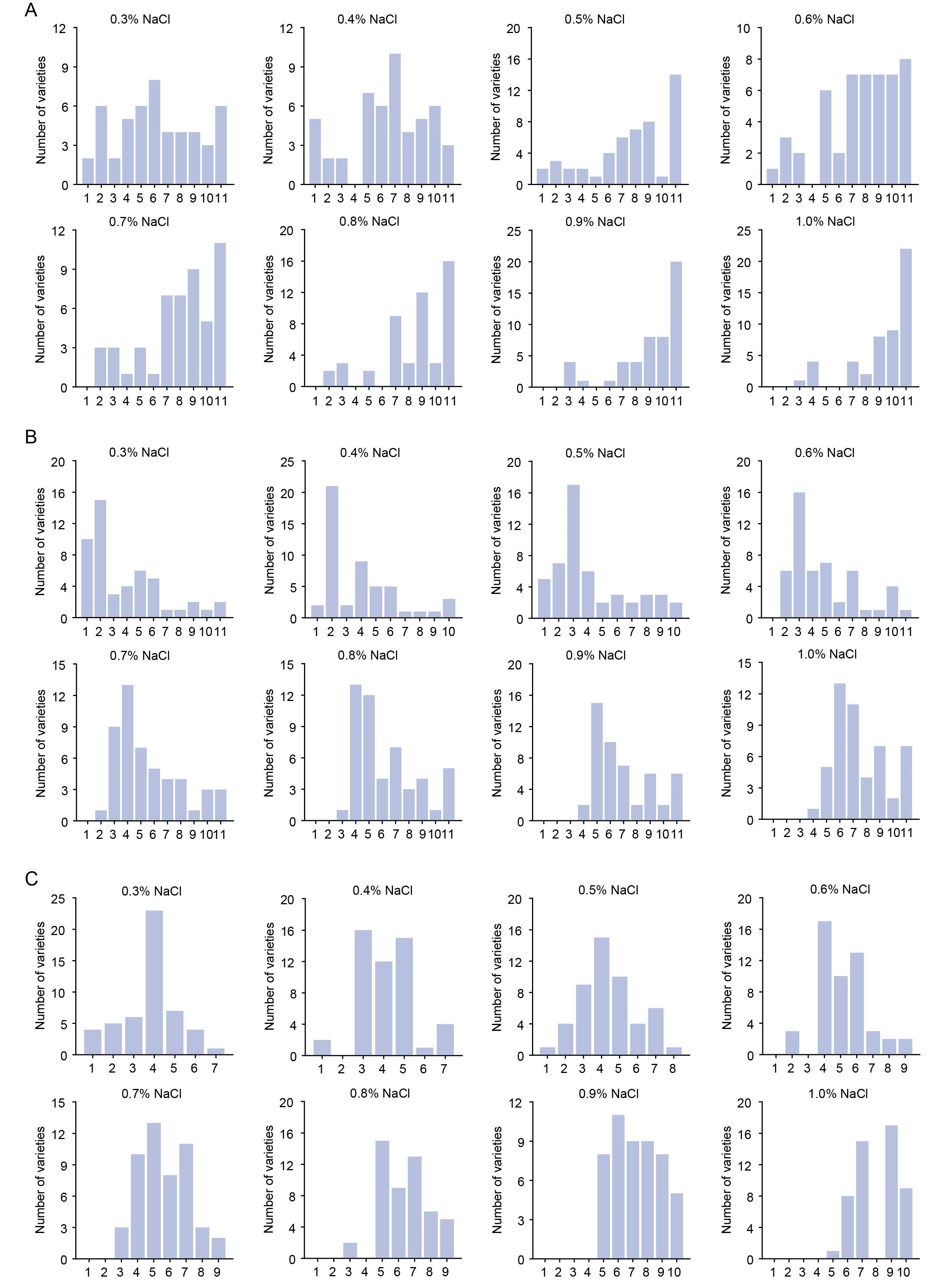

Supplement: Supplementary Figure 1 — Phenotypic variations of 50 randomly selected rice varieties for seed germination in NaCl concentrations from 0.3% to 1%. Germination rate grades on the 3rd (GRG3; A) and the 4th day (GRG4; B), and germination index grades (GIG; C) under the treatments if indicated NaCl concentrations. [file Image_1.jpeg]

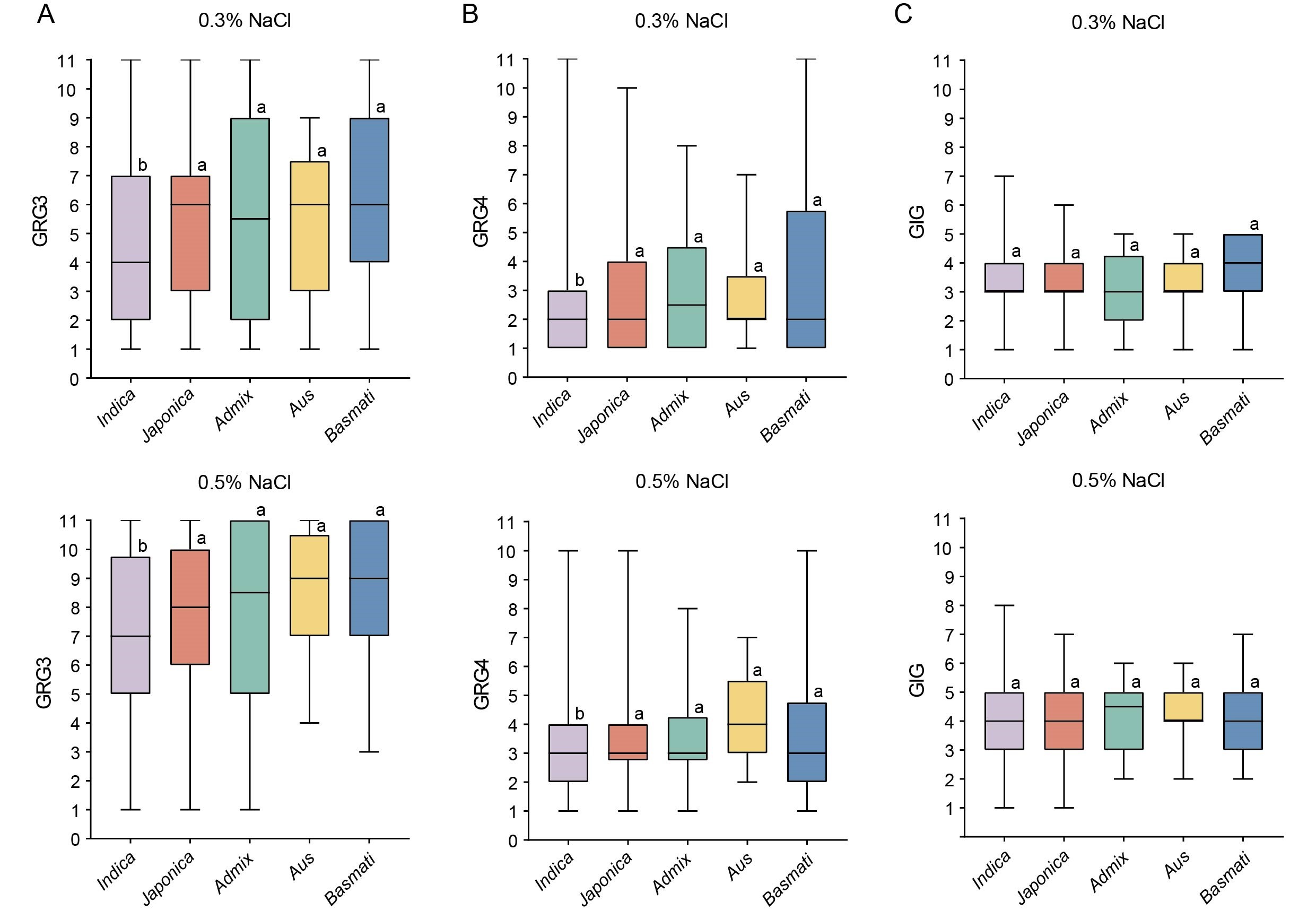

Supplement: Supplementary Figure 2 — Phenotypic variations for seed germinations under salt stresses among varieties in different subpopulations. (A) Germination rate grades on the 3rd day (GRG3) under 0.3% (top) and 0.5% NaCl (bottom) treatments in different subpopulations. (B) Germination rate grades on the 4th day (GRG4) under 0.3% (top) and 0.5% NaCl (bottom) conditions in different subpopulations. (C) Germination index grades (GIG) under the treatment of 0.3% (top) and 0.5% NaCl (bottom) in different subpopulations. Lowercase letters indicate significant differences based on Duncan’s multiple range post-hoc test (p < 0.05). [file Image_2.jpeg]

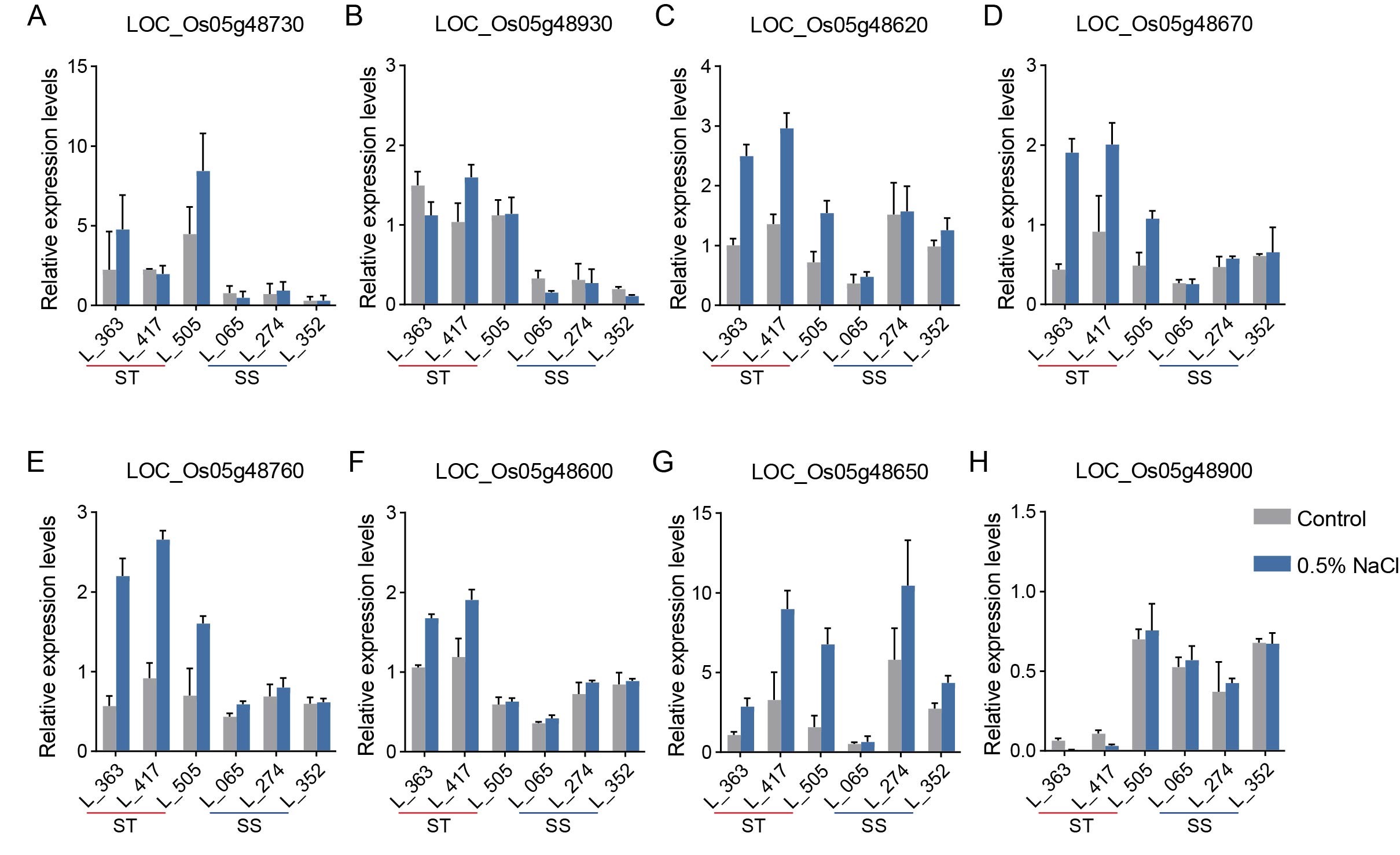

Supplement: Supplementary Figure 3 — Expressions of candidate genes in the qGRG3-2 locus in representative salt-tolerant and salt-sensitive varieties. ST, representative salt-tolerant varieties; SS, representative salt-sensitive varieties. [file Image_3.jpeg]
